# Robust regression for periodicity detection in non-uniformly sampled time-course gene expression data

**DOI:** 10.1186/1471-2105-8-233

**Published:** 2007-07-02

**Authors:** Miika Ahdesmäki, Harri Lähdesmäki, Andrew Gracey, llya Shmulevich, Olli Yli-Harja

**Affiliations:** 1 Institute of Signal Processing, Tampere University of Technology, P.O.Box 553, 33101 Tampere, Finland; 2 Institute for Systems Biology, WA 98103, USA; 3 Marine Environmental Biology, University of Southern California, CA 90089, USA

## Abstract

**Background:**

In practice many biological time series measurements, including gene microarrays, are conducted at time points that seem to be interesting in the biologist's opinion and not necessarily at fixed time intervals. In many circumstances we are interested in finding targets that are expressed periodically. To tackle the problems of uneven sampling and unknown type of noise in periodicity detection, we propose to use robust regression.

**Methods:**

The aim of this paper is to develop a general framework for robust periodicity detection and review and rank different approaches by means of simulations. We also show the results for some real measurement data.

**Results:**

The simulation results clearly show that when the sampling of time series gets more and more uneven, the methods that assume even sampling become unusable. We find that M-estimation provides a good compromise between robustness and computational efficiency.

**Conclusion:**

Since uneven sampling occurs often in biological measurements, the robust methods developed in this paper are expected to have many uses. The regression based formulation of the periodicity detection problem easily adapts to non-uniform sampling. Using robust regression helps to reject inconsistently behaving data points.

**Availability:**

The implementations are currently available for Matlab and will be made available for the users of R as well. More information can be found in the web-supplement [1].

## Background

The detection of periodically behaving gene expression time series has been an area of enormous interest lately. Since more and more microarray [2] data is becoming available, including time series, the periodicity detection methods from other branches of science are being modified for use in gene expression studies. Periodicity detection methods can be broadly divided into generic and more specific detection rules. The generic approaches use the available statistical theory to seek strong periodic components at all the available frequencies [3-7] and use exact tests to yield significance values with multiple correction. The more specific methods try to find periodic phenomena at specific frequencies, e.g. the assumed cell cycle frequency (see e.g. [8-15])

Some of the most severe problems of processing gene expression time series data include short time series length, the presence of noise of unknown distribution, outliers (i.e. points that are clearly inconsistent with most of the other points in the data), non-uniform sampling used in performing the experiments and other non-linearities involved in the measurement technologies themselves. Outliers can be thought of as low-probability values from a mixture model where with a high probability the noise in the signal is modelled by a (Gaussian) distribution and with a low probability by another distribution whose variance is much higher than that of the first one. In earlier work we presented a robust modification [5] of Fisher's *g*-test [6] for finding hidden periodicities in time series data. The method performs well both under the Gaussian noise assumption and when outliers and other non-linearities are present. However, non-uniform sampling, other than the one resulting from missing values, was not considered and the aim of this paper is to evaluate different robust methods for periodicity detection that can handle non-uniform sampling. Non-uniform sampling in periodicity detection has been previously considered in [7]. The authors use a so-called Lomb-Scargle periodogram to find the spectral estimate for a time series, not limited by non-uniform sampling, and then test whether the maximum value of the spectral estimate is significantly higher than the other values. While the method is mathematically sound and is based on an exact test, it is non-robust, as is the basic Fisher's test. The same issue applies to most of the other previously published methods. Exceptions to this are in [5] and in [15], where the authors use Bayesian detection and show that the method can handle data that is corrupted with uniform and Laplacian noise as well (besides Gaussian).

The matter of choosing the sampling scenario in a cost-effective way is discussed in [16] where the authors present an active learning based online algorithm for choosing the sampling strategy.

In [17] the authors have developed a periodicity detection method in which they fit orthogonal periodic polynomials to non-uniformly sampled data. If the periodicity of interest is not sinusoidal (e.g. narrow pulse signals), the method improves on the performance of the Lomb-Scargle periodogram, but reduces to it in the case of sinusoidal model, which is the case of interest for us. An approach based on similar ideas is presented in [18] where the authors use least squares fitting of wavelets which is especially suitable if we want to search for periodicity in non-uniformly sampled data with non-sinusoidal cyclic components.

In [19] the authors use the Lomb-Scargle periodogram for periodicity detection and show that it performs better than the combination of interpolation to uniform sampling and ordinary periodogram. They point out that there is a low-pass effect involved in interpolation that is a major problem. In [20] the authors use a complicated approach of neural networks for periodicity detection in non-uniformly sampled time series but use interpolation to uniform sampling first, which, according to [19], causes problems in the high frequency end of the spectra. In [21] the authors actually make use of non-uniform sampling in digital alias-free signal processing applications. However, their approach is based on the idea of being able to choose the sampling intervals, which is not the usual case in biological studies. A model similar to the one in this paper is presented in [22], where the authors aim to estimate a wide spectral range of frequencies of a non uniformly sampled signal. Their approach is, however, aimed more at real-time applications and longer signals than those usually present in microarray studies. Some of these methods can be thought to be improvements over the standard periodogram but non-robust when it comes to heavy tailed distributions and/or mixture models where the presence of (low probability) outliers can cause large residuals in the estimators and thus bias the results. The Bayesian approach [15] is a clear distinction to the aforementioned approaches and presents an opportunity to make use of prior knowledge, such as the frequency of the oscillation. It is shown in [15] that the Bayesian detector performs better than methods that assume a strict frequency of periodicity [9] in case the frequency is not exactly known a priori. Other Bayesian periodicity detectors are presented in [23-26].

In this paper we follow the general direction of Fisher's *g*-test together with multiple testing correction for the detection of periodic time series in multiple time series data. Several modifications are needed to take into account non-uniform sampling and unknown noise characteristics. We use several different robust regression based methods [27-30] to find the spectral estimate of a time series instead of using the basic non-robust periodogram. By using regression we can readily take non-uniform sampling into account.

After finding the spectral estimate we propose to replace the periodogram in the *g*-test with the robust spectral estimate. Since no analytical results for such modifications exist, we resort to permutation tests in finding the *p*-values. We also note that the test can be modified to yield a test for one specific chosen frequency if an a priori hypothesis is made about the frequency of the periodicity of interest.

To compare the performance of the different regression methods and some of the previously introduced novel methods [5,7,15] in this framework, we use simulations and show the receiver operating characteristic (ROC) figures under several noise and signal configurations and non-uniform sampling. The computational complexity of the different methods is also briefly considered.

As an application we choose one of the best performing methods and apply it to microarray data measured from the mussel *Mytilus californianus*. Our experiments indicate that there is no statistically significant connection between the circadian rhythm and cell cycle regulated genes that could be expected to show periodic expression.

## Results and discussion

### Simulation based performance

The performance of the different introduced estimators when using the *g*-test framework is now verified by simulations and through the use of receiver operating characteristic curves (ROC, see e.g. [31]). The following simulations are first based on the assumption that we do not know the frequency of the underlying periodic signal. In addition, we iteratively subtract the fitted sinusoidals in the process of calculating the Fourier coefficients when using the robust regression based methods (as suggested in [30]). We then simulate time series where we have approximate a priori knowledge on the frequency of periodicity and compare the performance of the regression approach to the rank based method presented in [5] and the Bayesian detector presented in [15].

**Figure 1:**
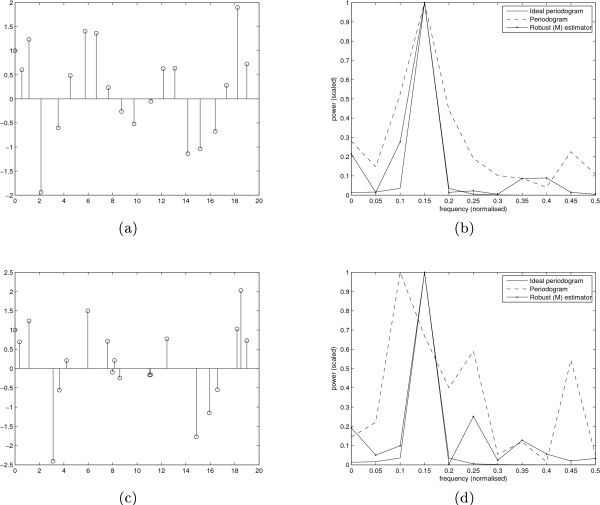
**Example signals**. Two example signals and their spectral estimates (scaled). The first simulated time series (a) is sampled according to the experimental mussel data. The sampling of the second time series (c) is an artificially deteriorated version of the first one. The corresponding spectral estimates, (b) and (d), include the ideal periodogram (Ideal periodogram), as if the time series was sampled uniformly and had no added noise, the periodogram of the samples (Periodogram). ignoring time indices, and the M-estimate (Robust (M) estimator).

Before going into the details of the ROC curves, we first present two non-uniformly sampled simulated periodic time series of length 20 and their estimated power spectra as examples. The periodic time series and the corresponding spectral estimates are shown in Figure 1. The sampling of the first time series (Figure 1a)) is chosen according to the experimental mussel data (Gracey et al., to appear in ArrayExpress [32]) that was conducted at time points 8.67, 10.52, 12.42, 15.67, 20.27, 23.52, 27.52, 30.52, 33.77, 37.32, 40.67, 45.22, 48.52, 51.67, 55.17, 58.47, 62.52, 65.52, 68.52 and 71.02 hours after the beginning of the experiment (see further description in subsection *The methods in practice*), so that the samples are taken usually approximately every three hours but sometimes there are four to five hour gaps. This sampling is a good representative of a real world measurement. The sampling of the second time series (Figure 1c)) is an artificially deteriorated version of the first one, so that sometimes the samples are closer together and sometimes farther apart. These artificial sample times were chosen to be 8.97, 10.12, 12.52, 18.67, 20.27, 22.12, 27.52, 32.52, 33.77, 34.32, 35.67, 43.22, 43.52, 47.67, 55.17, 58.47, 60.52, 65.52, 66.52 and 68.02 hours after the beginning of the experiment. The three spectral estimates in Figure 1b) and Figure 1d) correspond to an ideal spectral estimate (as if the sampling was uniform and there was no additive noise), the periodogram of the samples (ignoring time indices) and the regression based M-estimate. As we can see, in the first case (b) the periodogram estimate is still quite good but when the sampling gets worse (d), the periodogram no longer operates properly since it assumes uniform sampling. To test more formally which of the methods, if any, outperforms the others we now resort to the ROC curves.

The ROC curve is a plot of sensitivity (TPTP+FN)
 MathType@MTEF@5@5@+=feaafiart1ev1aaatCvAUfKttLearuWrP9MDH5MBPbIqV92AaeXatLxBI9gBaebbnrfifHhDYfgasaacH8akY=wiFfYdH8Gipec8Eeeu0xXdbba9frFj0=OqFfea0dXdd9vqai=hGuQ8kuc9pgc9s8qqaq=dirpe0xb9q8qiLsFr0=vr0=vr0dc8meaabaqaciaacaGaaeqabaqabeGadaaakeaadaqadaqaamaalaaabaGaemivaqLaemiuaafabaGaemivaqLaemiuaaLaey4kaSIaemOrayKaemOta4eaaaGaayjkaiaawMcaaaaa@3615@ on the y-axis, versus 1-specificity (FPFP+TN)
 MathType@MTEF@5@5@+=feaafiart1ev1aaatCvAUfKttLearuWrP9MDH5MBPbIqV92AaeXatLxBI9gBaebbnrfifHhDYfgasaacH8akY=wiFfYdH8Gipec8Eeeu0xXdbba9frFj0=OqFfea0dXdd9vqai=hGuQ8kuc9pgc9s8qqaq=dirpe0xb9q8qiLsFr0=vr0=vr0dc8meaabaqaciaacaGaaeqabaqabeGadaaakeaadaqadaqaamaalaaabaGaemOrayKaemiuaafabaGaemOrayKaemiuaaLaey4kaSIaemivaqLaemOta4eaaaGaayjkaiaawMcaaaaa@35F9@ on the *x*-axis. *TP *stands for true positive, *FN *for false negative and so on. For a perfect test the sensitivity is 1 for a specificity of 1. For any non-ideal tests the ROC curve shows the sensitivity-specificity tradeoff. The line segment from (0,0) to (1,1) is called the chance diagonal since the discrimination ability of a test is near to random if the curve is near to the diagonal.

#### A priori unknown frequency

We use the model of Equation (1) in the generation of 300 periodic time series corresponding to the alternative hypothesis and 300 nonperiodic time series corresponding to the null hypothesis. We consider the same two cases of non-uniform sampling described above. For each time series we calculate the *g*-statistic and order the values. We repetitively accept one more time series as periodic and since the ground truth is known, we can now tell the number of true and false positives and true and false negatives to construct the ROC curves for each of the methods. Since we assume the null distribution to be the same for all the time series we do not use permutation tests here. We chose to consider three types of noise for both of the non-uniform samplings, namely where the additive noise is Gaussian (std. 0.5,1.0), Gaussian (std. 0.75) with outliers at random locations (1,2 or 3 outliers, amplitude uniformly randomly chosen from ± (5 . . . 6)) or Laplacian (std. 1.0). The underlying periodic signal in the alternative hypothesis set always has an amplitude of 2
 MathType@MTEF@5@5@+=feaafiart1ev1aaatCvAUfKttLearuWrP9MDH5MBPbIqV92AaeXatLxBI9gBaebbnrfifHhDYfgasaacH8akY=wiFfYdH8Gipec8Eeeu0xXdbba9frFj0=OqFfea0dXdd9vqai=hGuQ8kuc9pgc9s8qqaq=dirpe0xb9q8qiLsFr0=vr0=vr0dc8meaabaqaciaacaGaaeqabaqabeGadaaakeaadaGcaaqaaiabikdaYaWcbeaaaaa@2DB9@ (the amplitude of the sum of zero phase cosine plus sine) as in [3] and the frequency of periodicity is uniformly random chosen in the interval [0.05,0.45]2*π*.

**Figure 2:**
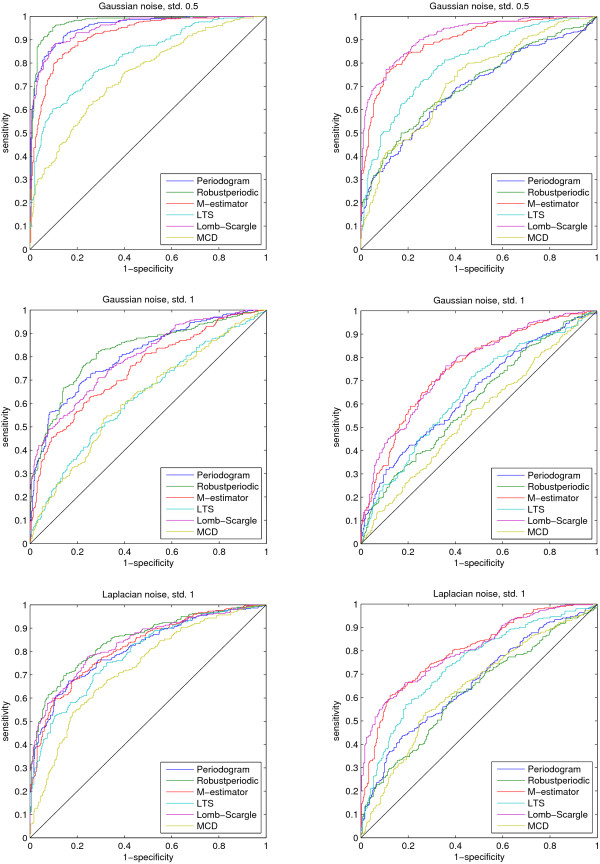
**Receiver operating characteristic curves 1**. The receiver operating characteristic curves for three different test cases and two sampling scenarios. On the left hand side the sampling is according to the mussel data while on the right hand side, the results for more deteriorated sampling are seen. The additive noise in this case is either Gaussian with varying standard deviation or Laplacian. The figure legends refer to the regression types except for Periodogram which is the ordinary periodogram ignoring time indices and Robustperiodic which corresponds to the method presented in [5].

**Figure 3:**
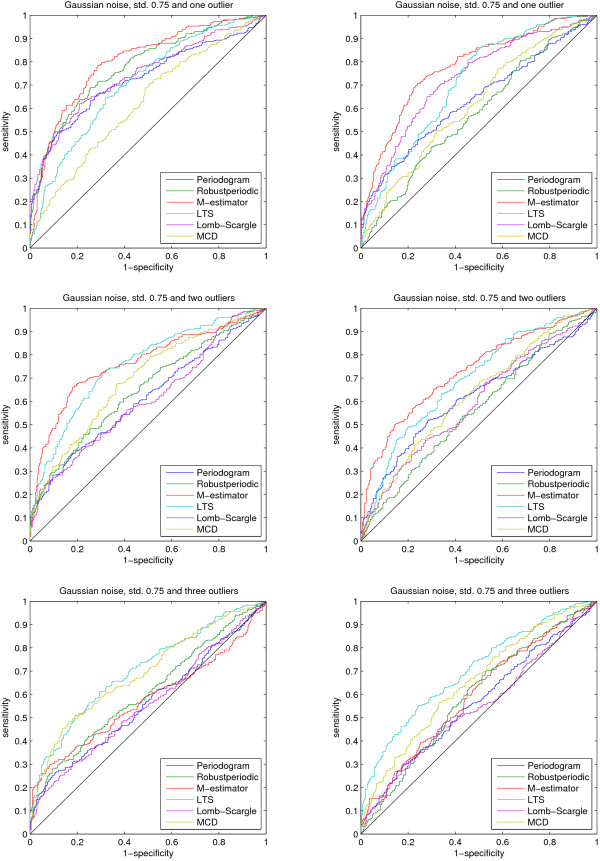
**Receiver operating characteristic curves 2**. The noise in this case is additive Gaussian with standard deviation of 0.75 and outliers of varying amount and amplitude. The figure legends refer to the same methods as in Figure 2.

Figure 2 shows that the robust method (denoted as Robustperiodic) we introduced in [5] performs the best in the case of the less non-uniform sampling and Gaussian or Laplacian noise. The ROC curves show that when the sampling is more non-uniform, the methods that assume uniform sampling no longer work at all. In these three cases the Lomb-Scargle periodogram and M-estimator based method still work rather well. Figure 3 clearly shows that outliers negatively affected performance of most of the methods. In the case of one or two outliers the M-estimator method outperforms the rest, but at 15% contamination (3 outliers, time series length 20) only the LTS based method can make a clear distinction from random guessing. It is discussed in [33,34] that any method that can not tolerate 10% of outliers should be used with caution. In addition, the methods that are not designed to handle non-uniform sampling quickly start to perform poorly the more random the sampling gets. It is interesting to note that the method introduced in [5] performs relatively well under the non-uniform sampling scenario of the mussel data although the method is not designed for non-uniform sampling.

#### A priori known frequency

In case we have prior knowledge on the frequency of the periodicity of interest, it is desirable to take this into account when trying to rank the time series according to periodicity. For the purpose of performance comparison we compare here the simulated ROC curves of three methods under the two presented sampling scenarios when the frequency is approximately known. We choose for the simulations the M-estimator as a representative of the regression estimators, the Bayesian approach method presented in [15] and the rank based method not designed for not-uniform sampling [5]. The strength of the Bayesian method [15] is that it is training-free and it does not assume a single frequency for the periodicity but rather a prior distribution whose mean and scale can be tuned according to prior knowledge.

We generate four sets of 300 time series according to the null hypothesis with Gaussian noise (std. 1) and 0, 1, 2 or 3 (0 in the time series of the first set, 1 in the second and so forth) randomly placed outliers of amplitude ± (5 . . . 6) per series. For the alternative hypothesis sets (4) we generate sinusoidals of random phase, amplitude 2
 MathType@MTEF@5@5@+=feaafiart1ev1aaatCvAUfKttLearuWrP9MDH5MBPbIqV92AaeXatLxBI9gBaebbnrfifHhDYfgasaacH8akY=wiFfYdH8Gipec8Eeeu0xXdbba9frFj0=OqFfea0dXdd9vqai=hGuQ8kuc9pgc9s8qqaq=dirpe0xb9q8qiLsFr0=vr0=vr0dc8meaabaqaciaacaGaaeqabaqabeGadaaakeaadaGcaaqaaiabikdaYaWcbeaaaaa@2DB9@ and frequency uniformly distributed in the range [0.09,0.11]2*π *in addition to the additive noise similar to the null hypothesis set. We then deliberately choose the frequency that is given to the periodicity detectors incorrectly as 2*π *0.125 (as in [15]) and observe the ROC curves for the three methods. The standard deviation for the Bayesian method was set to 0.1 as in [15].

**Figure 4:**
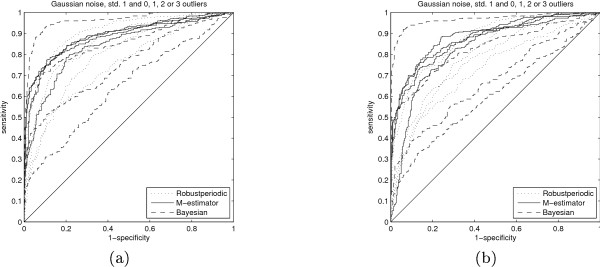
**Receiver operating characteristic curves 3**. The receiver operating characteristic curves for the two sampling scenarios, (a) according to the mussel data and (b) according to the deteriorated sampling, with prior knowledge on the frequency of the periodicity. The methods correspond to the rank based estimator (Robustperiodic), which does not take non-uniform sampling into account, Tukey's biweight regression estimator (M-estirnator) and the Bayesian method (Bayesian) presented in [15]. The frequency at which to look for periodicity is deliberately different from the true underlying frequency by approximately 25% to observe the effects of choosing the frequency incorrectly. In both (a) and (b) the effect of 1, 2 or 3 outliers is seen by the shift towards the chance diagonal (the closed to the chance diagonal corresponding to the 3-outlier case) from the case of no outliers.

**Figure 5:**
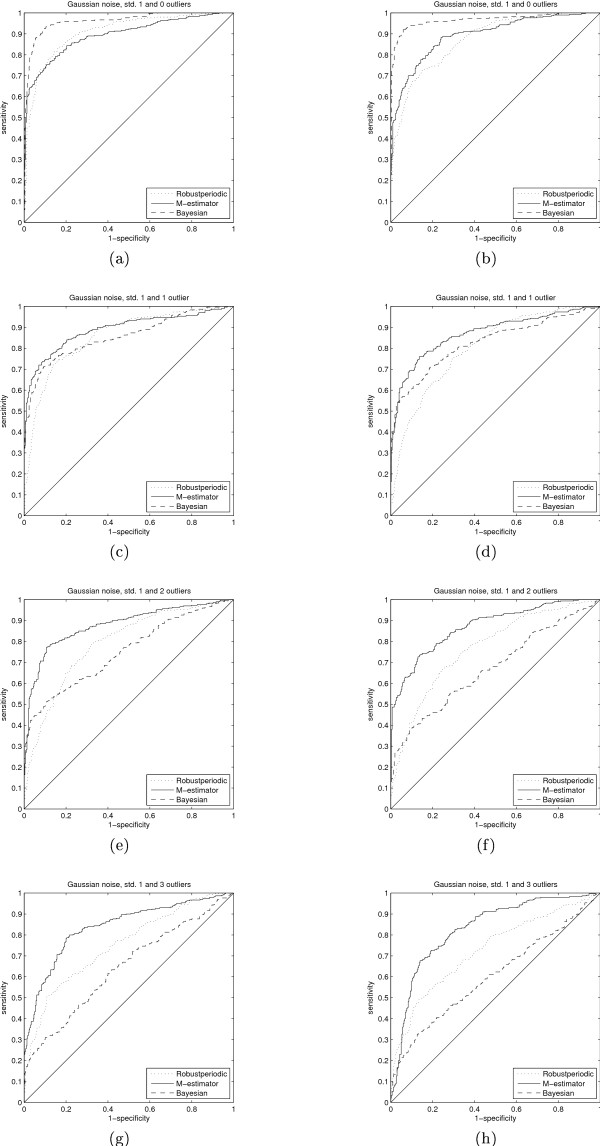
**Receiver operating characteristic curves 4**. This figure shows the data from Figure 4 but with the different noise cases separated.

As we can see in Figure 4a) and 4b) (alternative representation in Figure 5), the Bayesian method is superior to the other chosen periodicity detectors in case the additive noise present in the signals is pure Gaussian and our knowledge on the frequency is inaccurate. However, a single outlier changes the whole situation and, as can be seen, the performance of the M-estimator in case of 3 outliers (out of 20 samples), although suboptimal on Gaussian data and having chosen the frequency incorrectly, is approximately the same as the performance of the Bayesian method in case of one outlier. Thus, the M-estimator is much more robust when it comes to inconsistencies in the data.

As we can see, there is no single best approach to periodicity detection and one must weigh the options. If we want to reject outliers and have non-uniformly sampled data, the M-estimation based regression estimator is a safe choice. If we have a reason to trust in the Gaussian noise model (or other well behaving symmetric distribution) and we have weak prior knowledge on the frequency of the cycle period, the Bayesian approach [15] should be the best alternative. In the case of uniform sampling and no prior knowledge on the frequency, the rank based estimator [5] is also a considerable option, among others.

#### Computational complexity

Without going into more subtle considerations of asymptotic time complexity we just present the needed time to calculate the Fourier coefficients for two signal lengths using a modern laptop computer and Matlab. Although this running time comparison is not absolute and depends on particular implementations, it is indicative of general computational costs. In Table 1 we can see that the least trimmed squares (LTS) and minimum covariance determinant (MCS) regression methods take a lot of time if compared to the Lomb-Scargle and the Tukey's biweight M-estimator. In case of non-uniform sampling we therefore suggest to use the M-estimator since it performs quite fast and robustly as was seen in the subsection Simulation based performance. The Bayesian detector is not included in this comparison since it does not involve similar spectral estimation of the whole spectrum as the regression based methods here do. The implementation of the Bayesian detector is relatively slow if compared to the M-estimator when considering single frequency detection.

**Table 1:** Computational complexity. The approximate time in seconds needed for the different spectrum estimation methods to evaluate one spectral estimate. Random signals were used in the evaluation. FFT is the acronym for fast fourier transform, LTS for least trimmed squares and MCD for minimum ovariance determinant. The method Robustperiodic f fcorresponds to the method in [5]

	FFT	Robusst periodic	Lomb-Scargle	Tukey	LTS	MCD
Signal length						
20	0.00005	0.003	0.004	0.01	3.1	16.0
30	0.00004	0.004	0.004	0.15	5.0	22.3

### The methods in practice

We analyse here the microarray time series data set obtained from the mussel species *Mytilus californianus*, by measuring the gene expression over several days with non-uniform sampling. It is expected that the gene expression of some of the genes of the population would correlate with the tidal cycle or day and temperature cycle. We therefore seek the genes that are periodically expressed in the 24-hour cycle and show the annotations and time series profiles of the best ranking signals. In practice we use M-estimator based regression to fit sine and cosine signals of 24-hour cycle to the time series. To estimate the null hypothesis distribution (i.e. no periodicity present) for the estimated coefficients we use 300 random permutations of each time series.

**Figure 6:**
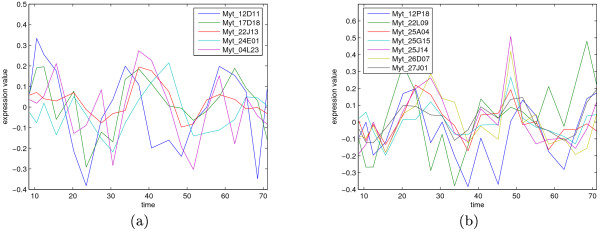
**The grouped periodic time series**. Two groups of periodic time series signals measured from the mussel Mytilus Californianus. The *x*-axis is time in hours and the first time point corresponds to 8:40 am. The approximately 24-hour cycle can be seen well. The figure legends show the gene names corresponding to the plotted time series.

Because we use a finite number of permutations in forming the null hypothesis distributions, *p*-values of zero (i.e. less than 1/300) are now possible. In fact, 12 of the best time series had a *p*-value of zero. The (grouped) best 12 time series are plotted in Figure 6 with the *x*-axis showing time in hours. As the first time point is at 8:40 am, we can see that there is a positive peak in (a) and a negative peak in (b) at around 12:00 am, and every 24 hours from then on. Since 12:00 am usually corresponds to the highest daily temperature, this suggests that these genes could have temperature (among other things) as a regulator. Two of the genes were not sequenced and seven of the genes were not annotated as of yet. The annotations for Myt_12D11, Myt_22J13 and Myt_12P18 are Transcription elongation factor B polypeptide 2 (RNA), Protein C3orf17 and Ubiquinol-cytochrome c reductase complex 7.2 kDa protein, correspondingly. As a comparison, we also applied the Bayesian detector [15] to the mussel data set. Forty genes were found that were in the list of top hundred ranked genes according to both of the methods, implying that the methods produce fairly similar results.

To determine how many of the best ranked genes we should accept as periodic we use the false discovery rate at a level of 0.1. This corresponds to 33 of the best time series out of 7679. This is quite a conservative decision boundary since the visual inspection of the first few hundred time series clearly shows similar patterns as in Figure 6.

The best detected genes were mostly expressed at small amplitude. To determine if there was a biological theme to the best detected genes, a Gene Set Enrichment Analysis (GSEA) [35] was performed using the list of genes ranked by the significance of periodic signal against 245 gene sets which were defined by their shared participation in a specific biological process in the Gene Ontology database. These gene sets covered biological processes which are known to be under circadian regulation in mammals such as the cell cycle and metabolic pathways. This analysis revealed that none of these gene sets were statistically significantly enriched towards the top of the ranked list of periodic genes. One explanation for this finding is that biological prior knowledge may be incomplete and does not capture the sets of genes that are under circadian regulation in mussels, or that the circadian cycle is attenuated in the tidal environment and does not really contribute much to the cyclic expression of the genes.

Replicate spots of each gene (that appear on the array) were removed prior to enrichment analysis to prevent false hits.

## Conclusion

The usual motivation for using methods based on least squares and a Gaussian model assumption, is that the theoretical background is thoroughly understood and computations are easier than in the case of robust methods. However, in reality, outlying data points are present in biological high throughput measurements such as microarrays. We have shown here that even a small number of outliers cause problems in periodicity detection if least squares methods are used. Taking into account the amount of data produced by microarray experiments, the manual replacing of these outliers is not possible and even if it was, the question of how to replace the outliers raises even more questions.

This leaves us with two reasonable approaches: we could either use a robust filter to clean the data first and then use methods based on least squares or use robust methods in the first place. If we however take a closer look at the filter cleaner proposed in [30], the method first evaluates a robust spectral estimate with M-estimator regression and then takes the inverse discrete Fourier transform to yield a cleaned time series. If we then evaluate the periodogram of the time series we end up with the same estimated power spectrum as we would have gotten by just using the M-estimator. This shows that in this case the two mentioned approaches are actually the same.

In addition to robustness against outliers we have shown in this paper that if sampling is not performed uniformly, it is very important to choose analysis methods that can adapt to not-uniform sampling. The regression based periodicity detection method implemented with the Tukey's biweight M-estimator was shown to have excellent performance in simulation studies and provided visually good results with real measurement data as well. Since the method can be readily implemented and is relatively fast to use, we propose it to be used as a first choice in periodicity detection with non-uniformly sampled data.

Future work includes the use of wavelets in periodicity detection. This has been previously considered in the literature in the context of spectrum estimation (e.g. [36]) and gene expression periodicity analysis [37] but it would be interesting to try to robustify the wavelet transform to reject outliers. Besides straightforward periodicity detection with wavelets, the robustify interpolation of the non-uniformly sampled time series to uniform sampling would be one reasonable approach as well. The ever growing need for data integration is also an urgent matter that must be given much attention in the future. Combining computational predictions from different data sources to increase statistical power is of utmost importance.

## Methods

The assumptions about the time series model and different methods for periodicity detection are first introduced. We then perform simulations and show the ROC curves for the methods when using the Fisher's *g*-statistic.

### Time series model

We use a similar (cyclo) stationary model for time series as in [3-5].

*y*_*n *_= *β *cos(ωn+ *φ*) + *ε*_*n*_,

where *β *≥ 0, *ω *∈ (0, *π*), *n *= 0,..., *N *- 1(∈ **Z**), *φ *∈ (-*π*, *π*], and *ε*_*n *_is an i.i.d. noise sequence (distribution unknown). To test for periodicity, define the null hypothesis as *H*_0_: *β *= 0, i.e., the time series consists of the noise sequence alone, *y*_*n *_= *ε*_*n*_.

To take non-uniform sampling into account we loosen the definition of Equation (1) so that *n *is replaced by *t*_*n*_. Time index *t*_*n *_can be any real number to allow the evaluation of the sine signal at arbitrary time points. For the practical implementation, see *Appendix A*.

### Periodicity detection

We first briefly review Fisher's test for the detection of periodic time series. We then generalise the test for different robust spectral estimators and finally we modify the test for the detection of a specific frequency.

#### Testing of all frequencies

Suppose we have a power spectral estimate *I*(*ω*). If the spectral estimate is obtained by using the classical periodogram (see e.g. [5,8] or [3]) at the (harmonic) normalised frequencies

ωl=2πlN,l=0,...,a
 MathType@MTEF@5@5@+=feaafiart1ev1aaatCvAUfKttLearuWrP9MDH5MBPbIqV92AaeXatLxBI9gBaebbnrfifHhDYfgasaacH8akY=wiFfYdH8Gipec8Eeeu0xXdbba9frFj0=OqFfea0dXdd9vqai=hGuQ8kuc9pgc9s8qqaq=dirpe0xb9q8qiLsFr0=vr0=vr0dc8meaabaqaciaacaGaaeqabaqabeGadaaakeaaiiGacqWFjpWDdaWgaaWcbaGaemiBaWgabeaakiabg2da9maalaaabaGaeGOmaiJae8hWdaNaemiBaWgabaGaemOta4eaaiabcYcaSiabdYgaSjabg2da9iabicdaWiabcYcaSiabc6caUiabc6caUiabc6caUiabcYcaSiabdggaHbaa@4049@

where *a *= [*N*/2] and the brackets denote the integer part of the rational number, then Fisher's *g*-statistic is defined as the maximum periodogram ordinate divided by the sum of all of the ordinates. Formally

g=max⁡1≤l≤qI(ωl)∑l=1qI(ωl),
 MathType@MTEF@5@5@+=feaafiart1ev1aaatCvAUfKttLearuWrP9MDH5MBPbIqV92AaeXatLxBI9gBaebbnrfifHhDYfgasaacH8akY=wiFfYdH8Gipec8Eeeu0xXdbba9frFj0=OqFfea0dXdd9vqai=hGuQ8kuc9pgc9s8qqaq=dirpe0xb9q8qiLsFr0=vr0=vr0dc8meaabaqaciaacaGaaeqabaqabeGadaaakeaacqWGNbWzcqGH9aqpdaWcaaqaaiGbc2gaTjabcggaHjabcIha4naaBaaaleaacqaIXaqmcqGHKjYOcqWGSbaBcqGHKjYOcqWGXbqCaeqaaOGaemysaKKaeiikaGccciGae8xYdC3aaSbaaSqaaiabdYgaSbqabaGccqGGPaqkaeaadaaeWaqaaiabdMeajjabcIcaOiab=L8a3naaBaaaleaacqWGSbaBaeqaaOGaeiykaKcaleaacqWGSbaBcqGH9aqpcqaIXaqmaeaacqWGXbqCa0GaeyyeIuoaaaGccqGGSaalaaa@4EAD@

where *q *= [(*N *- 1)/2] and we can analytically find the *p*-values for the statistic under the Gaussian assumption (see e.g. [8] or [6]). Other test statistics have been proposed for periodicity detection as well. For example, Chen [4] used a test called Hubert's C-test to complement the Fisher's test. The C-test tests whether a time series is a Gaussian white noise sequence. However, like Fisher's test, the C-test is based on the standard periodogram. Therefore, the C-test is not robust and even if the underlying sequence is Gaussian, a few outliers (e.g. measurement errors) can lead to incorrect results.

In [5] we developed a highly robust periodicity detection method that utilises the equivalence between the periodogram and correlogram spectral estimators. Robustness is obtained by replacing the standard autocorrelation estimator with a rank-based alternative. We used this robust spectral estimator instead of the periodogram in Equation (3). Since analytical results for the *p*-values of this modified distribution-free test do not exist (as of yet) we used permutation tests and simulated distributions to estimate the *p*-values. The rank-based method proposed in [5] does not, however, easily generalise to non-uniform sampling. In practice many biological time series measurements, including microarrays, are conducted at time points that are of biological interest and not necessarily at fixed time intervals. To tackle this problem, we propose to use robust regression based methods in the estimation of the spectral content. This way we can also take non-uniform sampling into account. Further motivation for this approach can be found by recalling that yet another (but still equivalent) formulation of the standard periodogram is obtained by using the frequency representation of a signal

yn=a0+∑i=1qa1icos⁡(ωitn)+∑j=1qa2jsin⁡(ωjtn)+(−1)naN/2,
 MathType@MTEF@5@5@+=feaafiart1ev1aaatCvAUfKttLearuWrP9MDH5MBPbIqV92AaeXatLxBI9gBaebbnrfifHhDYfgasaacH8akY=wiFfYdH8Gipec8Eeeu0xXdbba9frFj0=OqFfea0dXdd9vqai=hGuQ8kuc9pgc9s8qqaq=dirpe0xb9q8qiLsFr0=vr0=vr0dc8meaabaqaciaacaGaaeqabaqabeGadaaakeaacqWG5bqEdaWgaaWcbaGaemOBa4gabeaakiabg2da9iabdggaHnaaBaaaleaacqaIWaamaeqaaOGaey4kaSYaaabCaeaacqWGHbqydaWgaaWcbaGaeGymaeJaemyAaKgabeaakiGbcogaJjabc+gaVjabcohaZjabcIcaOGGaciab=L8a3naaBaaaleaacqWGPbqAaeqaaOGaemiDaq3aaSbaaSqaaiabd6gaUbqabaGccqGGPaqkaSqaaiabdMgaPjabg2da9iabigdaXaqaaiabdghaXbqdcqGHris5aOGaey4kaSYaaabCaeaacqWGHbqydaWgaaWcbaGaeGOmaiJaemOAaOgabeaakiGbcohaZjabcMgaPjabc6gaUjabcIcaOiab=L8a3naaBaaaleaacqWGQbGAaeqaaOGaemiDaq3aaSbaaSqaaiabd6gaUbqabaGccqGGPaqkcqGHRaWkcqGGOaakcqGHsislcqaIXaqmcqGGPaqkdaahaaWcbeqaaiabd6gaUbaaaeaacqWGQbGAcqGH9aqpcqaIXaqmaeaacqWGXbqCa0GaeyyeIuoakiabdggaHnaaBaaaleaacqWGobGtcqGGVaWlcqaIYaGmaeqaaOGaeiilaWcaaa@6E8D@

where the last term is omitted if *N *is odd. Parameters *a*_1*i *_and *a*_2*j *_now represent the frequency content of a time series signal. Under the commonly invoked i.i.d. Gaussian assumption, the optimal, i.e. minimum variance unbiased (MVU), estimates of the parameters can be obtained by using ordinary least squares regression (or simply inverse) to solve

y=[11⋮1A1A21−1⋮(−1)N−1][a0a1a2aN/2]
 MathType@MTEF@5@5@+=feaafiart1ev1aaatCvAUfKttLearuWrP9MDH5MBPbIqV92AaeXatLxBI9gBaebbnrfifHhDYfgasaacH8akY=wiFfYdH8Gipec8Eeeu0xXdbba9frFj0=OqFfea0dXdd9vqai=hGuQ8kuc9pgc9s8qqaq=dirpe0xb9q8qiLsFr0=vr0=vr0dc8meaabaqaciaacaGaaeqabaqabeGadaaakeaaieqacqWF5bqEcqGH9aqpdaWadaqaauaabeqabqaaaaqaauaabeqaeeaaaaqaaiabigdaXaqaaiabigdaXaqaaiabl6UinbqaaiabigdaXaaaaeaacqWGbbqqdaWgaaWcbaGaeGymaedabeaaaOqaaiabdgeabnaaBaaaleaacqaIYaGmaeqaaaGcbaqbaeqabqqaaaaabaGaeGymaedabaGaeyOeI0IaeGymaedabaGaeSO7I0eabaGaeiikaGIaeyOeI0IaeGymaeJaeiykaKYaaWbaaSqabeaacqWGobGtcqGHsislcqaIXaqmaaaaaaaaaOGaay5waiaaw2faamaadmaabaqbaeqabqqaaaaabaGaemyyae2aaSbaaSqaaiabicdaWaqabaaakeaacqWFHbqydaWgaaWcbaGae8xmaedabeaaaOqaaiab=fgaHnaaBaaaleaacqWFYaGmaeqaaaGcbaGaemyyae2aaSbaaSqaaiabd6eaojabc+caViabikdaYaqabaaaaaGccaGLBbGaayzxaaaaaa@53B7@

for [*a*_0 _**a**_**1 **_^*T *^**a**_**2 **_^*T *^*a*_*N*/2_]^*T*^. where **y **is the measured time series and again omitting *a*_*N*/2 _(and the last column in the matrix) if *N *is odd. Matrices *A*_1 _and *A*_2 _are defined as

A1=[cos⁡(ω1t0)⋯cos⁡(ωqt0)cos⁡(ω1t1)⋯cos⁡(ωqt1)⋮⋮cos⁡(ω1tN−1)⋯cos⁡(ωqtN−1)]
 MathType@MTEF@5@5@+=feaafiart1ev1aaatCvAUfKttLearuWrP9MDH5MBPbIqV92AaeXatLxBI9gBaebbnrfifHhDYfgasaacH8akY=wiFfYdH8Gipec8Eeeu0xXdbba9frFj0=OqFfea0dXdd9vqai=hGuQ8kuc9pgc9s8qqaq=dirpe0xb9q8qiLsFr0=vr0=vr0dc8meaabaqaciaacaGaaeqabaqabeGadaaakeaacqWGbbqqdaWgaaWcbaGaeGymaedabeaakiabg2da9maadmaabaqbaeqabqWaaaaabaGagi4yamMaei4Ba8Maei4CamNaeiikaGccciGae8xYdC3aaSbaaSqaaiabigdaXaqabaGccqWG0baDdaWgaaWcbaGaeGimaadabeaakiabcMcaPaqaaiabl+UimbqaaiGbcogaJjabc+gaVjabcohaZjabcIcaOiab=L8a3naaBaaaleaacqWGXbqCaeqaaOGaemiDaq3aaSbaaSqaaiabicdaWaqabaGccqGGPaqkaeaacyGGJbWycqGGVbWBcqGGZbWCcqGGOaakcqWFjpWDdaWgaaWcbaGaeGymaedabeaakiabdsha0naaBaaaleaacqaIXaqmaeqaaOGaeiykaKcabaGaeS47IWeabaGagi4yamMaei4Ba8Maei4CamNaeiikaGIae8xYdC3aaSbaaSqaaiabdghaXbqabaGccqWG0baDdaWgaaWcbaGaeGymaedabeaakiabcMcaPaqaaiabl6Uinbqaaaqaaiabl6UinbqaaiGbcogaJjabc+gaVjabcohaZjabcIcaOiab=L8a3naaBaaaleaacqaIXaqmaeqaaOGaemiDaq3aaSbaaSqaaiabd6eaojabgkHiTiabigdaXaqabaGccqGGPaqkaeaacqWIVlctaeaacyGGJbWycqGGVbWBcqGGZbWCcqGGOaakcqWFjpWDdaWgaaWcbaGaemyCaehabeaakiabdsha0naaBaaaleaacqWGobGtcqGHsislcqaIXaqmaeqaaOGaeiykaKcaaaGaay5waiaaw2faaaaa@8555@

A2=[sin⁡(ω1t0)⋯sin⁡(ωqt0)sin⁡(ω1t1)⋯sin⁡(ωqt1)⋮⋮sin⁡(ω1tN−1)⋯sin⁡(ωqtN−1)].
 MathType@MTEF@5@5@+=feaafiart1ev1aaatCvAUfKttLearuWrP9MDH5MBPbIqV92AaeXatLxBI9gBaebbnrfifHhDYfgasaacH8akY=wiFfYdH8Gipec8Eeeu0xXdbba9frFj0=OqFfea0dXdd9vqai=hGuQ8kuc9pgc9s8qqaq=dirpe0xb9q8qiLsFr0=vr0=vr0dc8meaabaqaciaacaGaaeqabaqabeGadaaakeaacqWGbbqqdaWgaaWcbaGaeGOmaidabeaakiabg2da9maadmaabaqbaeqabqWaaaaabaGagi4CamNaeiyAaKMaeiOBa4MaeiikaGccciGae8xYdC3aaSbaaSqaaiabigdaXaqabaGccqWG0baDdaWgaaWcbaGaeGimaadabeaakiabcMcaPaqaaiabl+UimbqaaiGbcohaZjabcMgaPjabc6gaUjabcIcaOiab=L8a3naaBaaaleaacqWGXbqCaeqaaOGaemiDaq3aaSbaaSqaaiabicdaWaqabaGccqGGPaqkaeaacyGGZbWCcqGGPbqAcqGGUbGBcqGGOaakcqWFjpWDdaWgaaWcbaGaeGymaedabeaakiabdsha0naaBaaaleaacqaIXaqmaeqaaOGaeiykaKcabaGaeS47IWeabaGagi4CamNaeiyAaKMaeiOBa4MaeiikaGIae8xYdC3aaSbaaSqaaiabdghaXbqabaGccqWG0baDdaWgaaWcbaGaeGymaedabeaakiabcMcaPaqaaiabl6Uinbqaaaqaaiabl6UinbqaaiGbcohaZjabcMgaPjabc6gaUjabcIcaOiab=L8a3naaBaaaleaacqaIXaqmaeqaaOGaemiDaq3aaSbaaSqaaiabd6eaojabgkHiTiabigdaXaqabaGccqGGPaqkaeaacqWIVlctaeaacyGGZbWCcqGGPbqAcqGGUbGBcqGGOaakcqWFjpWDdaWgaaWcbaGaemyCaehabeaakiabdsha0naaBaaaleaacqWGobGtcqGHsislcqaIXaqmaeqaaOGaeiykaKcaaaGaay5waiaaw2faaiabc6caUaaa@8677@

We should also verify that the above design matrix is invertible (full column rank), which should be the case unless the measurement times *t*_*n *_(or frequencies) are "pathological". To elaborate, for any frequency *ω *it is possible to choose *t*_*n *_so that, e.g., the cos term in one column of *A*_1 _is always equal to 1, making that column linearly dependent with the first column vector (vector of ones). It should, however, be unlikely that this happens in practice. Furthermore, given time instants *t*_*n*_, we can define the frequencies (perhaps slightly differently if needed) so that the columns are always linerly independent.

By choosing *ω *according to Equation (2) and assuming uniform sampling we can estimate the harmonic Fourier coefficients in **a**_**1 **_and **a**_**2**_. Evaluating

B(ω0)=N4(2a^0)2
 MathType@MTEF@5@5@+=feaafiart1ev1aaatCvAUfKttLearuWrP9MDH5MBPbIqV92AaeXatLxBI9gBaebbnrfifHhDYfgasaacH8akY=wiFfYdH8Gipec8Eeeu0xXdbba9frFj0=OqFfea0dXdd9vqai=hGuQ8kuc9pgc9s8qqaq=dirpe0xb9q8qiLsFr0=vr0=vr0dc8meaabaqaciaacaGaaeqabaqabeGadaaakeaacqWGcbGqcqGGOaakiiGacqWFjpWDdaWgaaWcbaGaeGimaadabeaakiabcMcaPiabg2da9maalaaabaGaemOta4eabaGaeGinaqdaaiabcIcaOiabikdaYiqbdggaHzaajaWaaSbaaSqaaiabicdaWaqabaGccqGGPaqkdaahaaWcbeqaaiabikdaYaaaaaa@3BD6@

B(ωl)=N4a^1l2+N4a^2l2,l=1,...,q
 MathType@MTEF@5@5@+=feaafiart1ev1aaatCvAUfKttLearuWrP9MDH5MBPbIqV92AaeXatLxBI9gBaebbnrfifHhDYfgasaacH8akY=wiFfYdH8Gipec8Eeeu0xXdbba9frFj0=OqFfea0dXdd9vqai=hGuQ8kuc9pgc9s8qqaq=dirpe0xb9q8qiLsFr0=vr0=vr0dc8meaabaqaciaacaGaaeqabaqabeGadaaakeaacqWGcbGqcqGGOaakiiGacqWFjpWDdaWgaaWcbaGaemiBaWgabeaakiabcMcaPiabg2da9maalaaabaGaemOta4eabaGaeGinaqdaaiqbdggaHzaajaWaa0baaSqaaiabigdaXiabdYgaSbqaaiabikdaYaaakiabgUcaRmaalaaabaGaemOta4eabaGaeGinaqdaaiqbdggaHzaajaWaa0baaSqaaiabikdaYiabdYgaSbqaaiabikdaYaaakiabcYcaSiabdYgaSjabg2da9iabigdaXiabcYcaSiabc6caUiabc6caUiabc6caUiabcYcaSiabdghaXbaa@4CCE@

B(ωN/2)=N4(2a^N/2)2
 MathType@MTEF@5@5@+=feaafiart1ev1aaatCvAUfKttLearuWrP9MDH5MBPbIqV92AaeXatLxBI9gBaebbnrfifHhDYfgasaacH8akY=wiFfYdH8Gipec8Eeeu0xXdbba9frFj0=OqFfea0dXdd9vqai=hGuQ8kuc9pgc9s8qqaq=dirpe0xb9q8qiLsFr0=vr0=vr0dc8meaabaqaciaacaGaaeqabaqabeGadaaakeaacqWGcbGqcqGGOaakiiGacqWFjpWDdaWgaaWcbaGaemOta4Kaei4la8IaeGOmaidabeaakiabcMcaPiabg2da9maalaaabaGaemOta4eabaGaeGinaqdaaiabcIcaOiabikdaYiqbdggaHzaajaWaaSbaaSqaaiabd6eaojabc+caViabikdaYaqabaGccqGGPaqkdaahaaWcbeqaaiabikdaYaaaaaa@3FF4@

yields a power spectral estimate *B*(*ω*) that coincides (see [38] p. 395) with the periodogram if the time series sampling is uniform. This explicitly shows that the periodogram can be obtained directly from a least squares estimate and is hence sensitive to different anomalies in data. On the other hand, the above regression based formulation opens up the possibility of developing alternative robust periodicity detection methods (and also robust spectrum estimation, see [30]) that can naturally handle non-uniform sampling. Since the dimensionality of the model matrix in Equation (5) is high and robust regression estimators do not necessarily yield optimum solutions easily for high dimensional models, we propose to find the parameters one frequency at a time. Therefore, for the purposes of robust estimation, we consider the following reduced model

**y **= *X*(*ω*)**b **+ **e**,

where **y **is the measured time series vector, **b **is the unknown parameter vector and **e **is a residual term. The matrix *X*(*ω*) is formed in the following way

X(ω)=[1cos⁡(ωt0)sin⁡(ωt0)1cos⁡(ωt1)sin⁡(ωt1)⋮⋮⋮1cos⁡(ωtN−1)sin⁡(ωtN−1)],
 MathType@MTEF@5@5@+=feaafiart1ev1aaatCvAUfKttLearuWrP9MDH5MBPbIqV92AaeXatLxBI9gBaebbnrfifHhDYfgasaacH8akY=wiFfYdH8Gipec8Eeeu0xXdbba9frFj0=OqFfea0dXdd9vqai=hGuQ8kuc9pgc9s8qqaq=dirpe0xb9q8qiLsFr0=vr0=vr0dc8meaabaqaciaacaGaaeqabaqabeGadaaakeaacqWGybawcqGGOaakiiGacqWFjpWDcqGGPaqkcqGH9aqpdaWadaqaauaabeqaemaaaaqaaiabigdaXaqaaiGbcogaJjabc+gaVjabcohaZjabcIcaOiab=L8a3jabdsha0naaBaaaleaacqaIWaamaeqaaOGaeiykaKcabaGagi4CamNaeiyAaKMaeiOBa4MaeiikaGIae8xYdCNaemiDaq3aaSbaaSqaaiabicdaWaqabaGccqGGPaqkaeaacqaIXaqmaeaacyGGJbWycqGGVbWBcqGGZbWCcqGGOaakcqWFjpWDcqWG0baDdaWgaaWcbaGaeGymaedabeaakiabcMcaPaqaaiGbcohaZjabcMgaPjabc6gaUjabcIcaOiab=L8a3jabdsha0naaBaaaleaacqaIXaqmaeqaaOGaeiykaKcabaGaeSO7I0eabaGaeSO7I0eabaGaeSO7I0eabaGaeGymaedabaGagi4yamMaei4Ba8Maei4CamNaeiikaGIae8xYdCNaemiDaq3aaSbaaSqaaiabd6eaojabgkHiTiabigdaXaqabaGccqGGPaqkaeaacyGGZbWCcqGGPbqAcqGGUbGBcqGGOaakcqWFjpWDcqWG0baDdaWgaaWcbaGaemOta4KaeyOeI0IaeGymaedabeaakiabcMcaPaaaaiaawUfacaGLDbaacqGGSaalaaa@7F74@

where *ω *is defined similarly as in Equation (1) and *t*_*n *_is the real-valued index.

To estimate **b **at the different frequencies we propose to use robust regression methods. The different robust regression estimators we chose for evaluation are reviewed in subsection *The different estimators*. It is suggested in [30] that when calculating the coefficients one frequency at a time, the fitted sinusoidal of the latest iteration should be subtracted from the signal **y **(i.e. leaving the residual). This residual is then used in place of **y **when estimating the parameter **b **of the next frequency, thus avoiding the problems caused by the loss of orthogonality. In addition, the order in which the frequencies should be sought is based on an initial spectral estimate, which is obtained by regression of the coefficients without any subtraction. The strategy is then to process the frequencies according to the magnitude of the coefficients, in descending order. To expand, in the case of uniform sampling, sinusoidals at the Fourier frequencies constitute an orthonormal basis. Thus in the least squares case, it is irrelevant in which order we fit the sinusoidals, since they are independent and the resulting spectral estimate will be the same [8]. If we have non-uniform sampling or we use estimators other than least squares we can no longer use this property.

The dependence between the fitted sinusoidals and the problem of overfitting can be corrected, at least to a reasonably good degree [30], by removing the fitted signal and fitting to the residuals. We should also mention that the non-orthogonolity would not be a problem if all the sinusoidals were fitted simultaneously, but this is not a reasonable approach for the robust estimators (due to the problems of high dimensionality and non-convergence.

We can now use the robust power spectral estimate *B*(*ω*) in Equation (3) without the requirement of uniform time series sampling. However, if the time series sampling is not uniform, the notions of Nyquist frequency and harmonic Fourier frequencies are no longer clear. We can still approximate the sampling frequency as if the sampling was uniform, as explained in *Appendix A*, and thus approximate the Fourier frequencies as well. For more discussion on the subject, see [39]. *p*-values can be estimated using simulated distributions or permutation tests. The use of simulated distributions is now more awkward than in the case of the rank-based method of [**5**]. It was noted in [5] that the rank-based method is "distribution-free", which implies that it is sufficient to simulate the null hypothesis distribution for only one noise type (e.g., Gaussian) under the assumption that the noise is i.i.d. The proposed regression based methods are not distribution-free and we would have to generate the null hypothesis distribution for all the different imaginable noise types separately. Of course, independent of the method, the null hypothesis distribution must be generated separately for different time series lengths. Therefore, based on the preceding discussion we propose to use permutation tests with multiple correction method of Benjamini and Hochberg [40] in the same way as in [5] to find the significance values.

#### Testing of one frequency

If we have *a priori *information about the frequency of the interesting periodic phenomenon, we do not need to seek periodicities at all the frequencies. There are different ways of modifying Equation (3) to take this additional information into account. In [5] we replaced max_1 ≤ *l *≤ *a*_*I *(*ω*_l_) with just the power spectral estimate at the index of interest to concentrate on just the interesting frequency. If we want to extend our search to other than the harmonic Fourier frequencies then the denominator in Equation (3) loses its meaning. Therefore we propose to use the following modified statistic

gm=B(ωc)=b^1c2+b^2c2,
 MathType@MTEF@5@5@+=feaafiart1ev1aaatCvAUfKttLearuWrP9MDH5MBPbIqV92AaeXatLxBI9gBaebbnrfifHhDYfgasaacH8akY=wiFfYdH8Gipec8Eeeu0xXdbba9frFj0=OqFfea0dXdd9vqai=hGuQ8kuc9pgc9s8qqaq=dirpe0xb9q8qiLsFr0=vr0=vr0dc8meaabaqaciaacaGaaeqabaqabeGadaaakeaacqWGNbWzdaWgaaWcbaGaemyBa0gabeaakiabg2da9iabdkeacjabcIcaOGGaciab=L8a3naaBaaaleaacqWGJbWyaeqaaOGaeiykaKIaeyypa0JafmOyaiMbaKaadaqhaaWcbaGaeGymaeJaem4yamgabaGaeGOmaidaaOGaey4kaSIafmOyaiMbaKaadaqhaaWcbaGaeGOmaiJaem4yamgabaGaeGOmaidaaOGaeiilaWcaaa@430E@

where *g*_*m *_is the modified *g*-statistic, *ω*_*c *_is the frequency of interest and b^1c2
 MathType@MTEF@5@5@+=feaafiart1ev1aaatCvAUfKttLearuWrP9MDH5MBPbIqV92AaeXatLxBI9gBaebbnrfifHhDYfgasaacH8akY=wiFfYdH8Gipec8Eeeu0xXdbba9frFj0=OqFfea0dXdd9vqai=hGuQ8kuc9pgc9s8qqaq=dirpe0xb9q8qiLsFr0=vr0=vr0dc8meaabaqaciaacaGaaeqabaqabeGadaaakeaacuWGIbGygaqcamaaDaaaleaacqaIXaqmcqWGJbWyaeaacqaIYaGmaaaaaa@3167@ and b^2c2
 MathType@MTEF@5@5@+=feaafiart1ev1aaatCvAUfKttLearuWrP9MDH5MBPbIqV92AaeXatLxBI9gBaebbnrfifHhDYfgasaacH8akY=wiFfYdH8Gipec8Eeeu0xXdbba9frFj0=OqFfea0dXdd9vqai=hGuQ8kuc9pgc9s8qqaq=dirpe0xb9q8qiLsFr0=vr0=vr0dc8meaabaqaciaacaGaaeqabaqabeGadaaakeaacuWGIbGygaqcamaaDaaaleaacqaIYaGmcqWGJbWyaeaacqaIYaGmaaaaaa@3169@ correspond to the coefficients of the fitted sine and cosine terms (Equation 11) at this frequency. The first problem arises with *p*-value computation. If we use ordinary least squares (OLS) for the regression then we can just neglect the denominator in Equation (3) since, as will be explained later, we will be using permutation tests for *p*-value computation and the denominator is the same for all the permutations of a single time series; thus, there is no need for the scaling. However when using the robust regression based methods there is no guarantee that the sum of the terms is a constant for different permutations. On the other hand, in many cases the effect of the omission of the scaling factor in the denominator has little effect on periodicity detection. This is also verified with good results on real data in subsection *The methods in practice*. Moreover, this way we do not need to compute the whole spectral estimate but just at the frequency of interest, which can now be other than one of the harmonic Fourier frequencies as well. This is a huge benefit for the use of the regression methods since the robust ones are typically computationally time-consuming. To sum up, we propose to use the following procedure for finding periodicities at a known frequency:

1. For a time series, fit the model of Equation (11) at a chosen frequency *ω*_*c*_

2. Evaluate Equation (13).

3. Randomly permute the original time series and for each permutation repeat steps 1 and 2.

4. To estimate the null hypothesis distribution of the modified *g*-statistic, compose a histogram of the population of *g*-statistics generated in step 3.

5. Use the histogram as a distributional estimate to get a *p*-value for the original test statistic computed in step 2.

6. Repeat steps 1–5 for all the time series to get the necessary *p*-values.

7. use multiple correction for the obtained *p*-values.

Although the idea of robustly fitting sinusoidals of one frequency to data is not new. we have here plausibly incorporated it for periodicity detection by modifying Fisher's test. We would like to note that exact tests are also available for some robust tests (see e.g. [27]). However, since these tests require some knowledge of the underlying distributions and because they are not applicable to all robust methods considered here, we propose to use the general purpose non-parametric permutation tests.

### The different estimators

In this subsection we present the different estimators that we will use in periodicity detection. We will mainly concentrate on robust estimators but will also present for comparison purposes the non-robust Lornb-Scargle periodogram, a modification of the ordinary periodogram taking non-uniform sampling into account.

#### M-estimators – the Tukey's biweight

We consider the popular redescending M-estimators which are able to reject outliers in the response variables entirely. Since the measurement time points are typically known, it is reasonable to assume that the regressors (i.e. the time points) are deterministic, a priori known and not random variables. Tatum and Hurvich [30] used the M-estimator with Turkey's biweight as the cost function to yield a robust time series filter that performed well with both simulated and real data. The central idea behind the filter is to first evaluate a robust spectral estimate of the time series and then use inverse FFT to estimate the clean time series. We therefore opt to use the biweight as a representative M-estimator in our periodicity detection study. It should be noted that the ordinary least squares is a special case of M-estimators. The objective of M-estimators is to find

min⁡b∑n=0N−1ρ(rnσn),
 MathType@MTEF@5@5@+=feaafiart1ev1aaatCvAUfKttLearuWrP9MDH5MBPbIqV92AaeXatLxBI9gBaebbnrfifHhDYfgasaacH8akY=wiFfYdH8Gipec8Eeeu0xXdbba9frFj0=OqFfea0dXdd9vqai=hGuQ8kuc9pgc9s8qqaq=dirpe0xb9q8qiLsFr0=vr0=vr0dc8meaabaqaciaacaGaaeqabaqabeGadaaakeaadaWfqaqaaiGbc2gaTjabcMgaPjabc6gaUbWcbaacbeGae8NyaigabeaakmaaqahabaacciGae4xWdihaleaacqWGUbGBcqGH9aqpcqaIWaamaeaacqWGobGtcqGHsislcqaIXaqma0GaeyyeIuoakmaabmaabaWaaSaaaeaacqWGYbGCdaWgaaWcbaGaemOBa4gabeaaaOqaaiab+n8aZnaaBaaaleaacqWGUbGBaeqaaaaaaOGaayjkaiaawMcaaiabcYcaSaaa@45AA@

where *ρ *is a symmetric function with a unique minimum at zero, *r*_*n *_= *y*_*n *_- xnT
 MathType@MTEF@5@5@+=feaafiart1ev1aaatCvAUfKttLearuWrP9MDH5MBPbIqV92AaeXatLxBI9gBaebbnrfifHhDYfgasaacH8akY=wiFfYdH8Gipec8Eeeu0xXdbba9frFj0=OqFfea0dXdd9vqai=hGuQ8kuc9pgc9s8qqaq=dirpe0xb9q8qiLsFr0=vr0=vr0dc8meaabaqaciaacaGaaeqabaqabeGadaaakeaaieaacqWF4baEdaqhaaWcbaGaemOBa4gabaGaemivaqfaaaaa@30ED@**b **is the residual of the *n*^th ^datum, xnT
 MathType@MTEF@5@5@+=feaafiart1ev1aaatCvAUfKttLearuWrP9MDH5MBPbIqV92AaeXatLxBI9gBaebbnrfifHhDYfgasaacH8akY=wiFfYdH8Gipec8Eeeu0xXdbba9frFj0=OqFfea0dXdd9vqai=hGuQ8kuc9pgc9s8qqaq=dirpe0xb9q8qiLsFr0=vr0=vr0dc8meaabaqaciaacaGaaeqabaqabeGadaaakeaaieaacqWF4baEdaqhaaWcbaGaemOBa4gabaGaemivaqfaaaaa@30ED@ = [1, cos(*ωt*_*n*_), sin(*ωt*_*n*_)], and *σ*_*n *_are scaling factors. The scaling factors *σ*_*n *_are chosen so that the resulting estimator is approximately 95% as efficient as the least squares estimator when applied to normally distributed data with no outliers. In particular, *σ*_*n *_= 4.685·s^1−hn
 MathType@MTEF@5@5@+=feaafiart1ev1aaatCvAUfKttLearuWrP9MDH5MBPbIqV92AaeXatLxBI9gBaebbnrfifHhDYfgasaacH8akY=wiFfYdH8Gipec8Eeeu0xXdbba9frFj0=OqFfea0dXdd9vqai=hGuQ8kuc9pgc9s8qqaq=dirpe0xb9q8qiLsFr0=vr0=vr0dc8meaabaqaciaacaGaaeqabaqabeGadaaakeaacuWGZbWCgaqcamaakaaabaGaeGymaeJaeyOeI0IaemiAaG2aaSbaaSqaaiabd6gaUbqabaaabeaaaaa@3302@ is used for each *n*, where *ŝ *= 1.4826 mad{*r*_*i*_} is the scaled median absolute deviation of the residuals from their median and *h*_*n *_= (*X*(*X*^*T*^*X*)-^1^*X*^*T*^)_*nn*_, i.e, the *n*^th ^diagonal element of the "hat" matrix. For further details, see [41] and implementation details of robustfit function in [42]. In the search for the solution of Equation (14) it is useful to solve for the stationary point by setting

∑n=0N−1σn−1ψ(rnσn)xnT=0,
 MathType@MTEF@5@5@+=feaafiart1ev1aaatCvAUfKttLearuWrP9MDH5MBPbIqV92AaeXatLxBI9gBaebbnrfifHhDYfgasaacH8akY=wiFfYdH8Gipec8Eeeu0xXdbba9frFj0=OqFfea0dXdd9vqai=hGuQ8kuc9pgc9s8qqaq=dirpe0xb9q8qiLsFr0=vr0=vr0dc8meaabaqaciaacaGaaeqabaqabeGadaaakeaadaaeWbqaaGGaciab=n8aZnaaDaaaleaacqWGUbGBaeaacqGHsislcqaIXaqmaaaabaGaemOBa4Maeyypa0JaeGimaadabaGaemOta4KaeyOeI0IaeGymaedaniabggHiLdGccqWFipqEdaqadaqaamaalaaabaGaemOCai3aaSbaaSqaaiabd6gaUbqabaaakeaacqWFdpWCdaWgaaWcbaGaemOBa4gabeaaaaaakiaawIcacaGLPaaaieqacqGF4baEdaqhaaWcbaGaemOBa4gabaGaemivaqfaaOGaeyypa0Jae4hmaaJaeiilaWcaaa@4B59@

where the influence function *ψ *(Tukey's biweight function in this case) is the derivative of *ρ*. The idea of the biweight M-estimator is to give zero weighting to those data points whose residuals are large if compared to the estimated scale. Therefore, if the scale is estimated robustly as well, we expect the M-estimators to give us good performance on data with different distributional characteristics. For more information on M-estimators, see e.g. [27,41]. In our computations we use the implementation in Matlab's Statistics Toolbox that uses iteratively reweighted least squares estimation.

#### Least trimmed squares (LTS)

The least trimmed squares regression [28] (LTS) is a robust regression method that was developed to have similar robustness properties as the least median of squares (LMS) regression but that would perform better under the Gaussian assumption and would be less computationally expensive.

The central idea behind the LTS is that instead of considering complete sets we choose subsets of the measured time points of the variables and use OLS for each of these subsets. We then choose the estimate that yields the smallest residual variance. Quantitatively, order the residuals of the linear model as (*r*^2^)_1 _≤ (*r*^2^)_2 _≤ ... ≤ (*r*^2^)_*N*_. Then the LTS is defined as

min⁡b∑i=1h(r2)i,
 MathType@MTEF@5@5@+=feaafiart1ev1aaatCvAUfKttLearuWrP9MDH5MBPbIqV92AaeXatLxBI9gBaebbnrfifHhDYfgasaacH8akY=wiFfYdH8Gipec8Eeeu0xXdbba9frFj0=OqFfea0dXdd9vqai=hGuQ8kuc9pgc9s8qqaq=dirpe0xb9q8qiLsFr0=vr0=vr0dc8meaabaqaciaacaGaaeqabaqabeGadaaakeaadaWfqaqaaiGbc2gaTjabcMgaPjabc6gaUbWcbaacbeGae8NyaigabeaakmaaqahabaGaeiikaGIaemOCai3aaWbaaSqabeaacqaIYaGmaaGccqGGPaqkdaWgaaWcbaGaemyAaKgabeaaaeaacqWGPbqAcqGH9aqpcqaIXaqmaeaacqWGObaAa0GaeyyeIuoakiabcYcaSaaa@3FFE@

where [*N*/2] + 1 ≤ *h *≤ *N*. In practice not all subsets can be considered and only some hundreds or maybe thousands of randomly chosen subsets are processed to yield the output.

One important feature of the LTS (and the MCD, which will be introduced shortly) regression is that it can tolerate outliers in the predictor variables as well, whereas the M-estimators cannot. However, this is not critical in spectrum estimation since the measurement time points usually contain no stochasticity. The implementation (FAST-LTS) is introduced in [43].

#### Minimum covariance determinant

The minimum covariance determinant (MCD) regression method [29] is a well performing robust regression method that can also handle cases where both *X *and *y *are multivariate. This method has similarities to the LTS regression in that it also considers subsets instead of complete sets in the estimation. The subset that yields the smallest covariance determinant is chosen for the estimation. For multivariate regression the model is presented as

*y *= *β*^*T*^*X *+ *α *+ *ε*,

where *y *is *q*-variate, *X *is *p*-variate, *β *is the (*p *× *q*] slope matrix, *α *is the *q*-dimensisnal intercept vector and *ε *is i.i.d with zero mean and with a positive definite covariance matrix.

Since the standard LS estimates of *β *and *α *can be written as functions of the estimated empirical mean vector (location) and the covariance matrix (scatter), a logical way of robustifying the regression is to replace the location vector and the scatter matrix with robust alternatives. In [29] the authors use the minimum covariance determinant estimator to yield the necessary robust estimators for the location and covariance matrix, thus leading to robust regression. It is shown that this is a positive-breakdown and bounded-influence method, which, if properly reweighted and iterated, has a high efficiency. The implementation of the algorithm (FAST-MCD) is introduced in [44].

#### The Lomb-Scargle periodogram

The Lomb-Scargle periodogram (see e.g. [7]) can be seen as a least squares fit of unequally sampled sinusoids to the measured signal and thus an extension of the periodogram. Exact tests for periodicity detection have been developed but problems include the well known outlier sensitivity of the OLS [45].

#### A note on missing values

It frequently occurs that besides non-uniform sampling there are some measurement points in some genes that are missing although they are present in other genes, thus reducing the quality of the data. Since we do not use an analytical null hypothesis distribution for all the time series but rather simulate the distribution for each time series separately, these missing points are handled so that we fit the sinusoidals only to the time points that are present. The presented methods thus have no further need for missing value imputation (a topic which has received considerable attention lately, see e.g. [46]).

## Appendix A. Supplementary information

To take non-uniform sampling into account, we loosen the definition of the variable *n *in Equation (1) so that it does not need to be integer-valued. We perform a scaling of the measurement time points in the following way: Denote the time points when the actual measurements have been made as a vector

*τ*= [*τ*_0_, ..., *τ*_*N *- 1_]^*T*^.

Then form the vector

t=(τ¯−τ0⋅1)(N−1)τN−1−τ0
 MathType@MTEF@5@5@+=feaafiart1ev1aaatCvAUfKttLearuWrP9MDH5MBPbIqV92AaeXatLxBI9gBaebbnrfifHhDYfgasaacH8akY=wiFfYdH8Gipec8Eeeu0xXdbba9frFj0=OqFfea0dXdd9vqai=hGuQ8kuc9pgc9s8qqaq=dirpe0xb9q8qiLsFr0=vr0=vr0dc8meaabaqaciaacaGaaeqabaqabeGadaaakeaaieqacqWF0baDcqGH9aqpdaWcaaqaaiabcIcaOGGaciqb+r8a0zaaDaGaeyOeI0Iae4hXdq3aaSbaaSqaaiabicdaWaqabaGccqGHflY1cqWFXaqmcqGGPaqkcqGGOaakcqWGobGtcqGHsislcqaIXaqmcqGGPaqkaeaacqGFepaDdaWgaaWcbaGaemOta4KaeyOeI0IaeGymaedabeaakiabgkHiTiab+r8a0naaBaaaleaacqaIWaamaeqaaaaaaaa@474C@

to correspond to the new indices, i.e. we normalise the last time point to *N *- 1. Note that for any uniformly sampled time series Eq. (19) yields an integer valued vector **t**.

To find the corresponding real time frequency for an angular frequency *ω *∈ (0, *π*) we consider two interpretations of the *ω*. The first one is in Equation (2) and the second one is

*ω *= 2*πf*/*F*_*s*_,

where *f *is the frequency of periodicity and *F*_*s *_is the sampling frequency. Therefore

f=FslN.
 MathType@MTEF@5@5@+=feaafiart1ev1aaatCvAUfKttLearuWrP9MDH5MBPbIqV92AaeXatLxBI9gBaebbnrfifHhDYfgasaacH8akY=wiFfYdH8Gipec8Eeeu0xXdbba9frFj0=OqFfea0dXdd9vqai=hGuQ8kuc9pgc9s8qqaq=dirpe0xb9q8qiLsFr0=vr0=vr0dc8meaabaqaciaacaGaaeqabaqabeGadaaakeaacqWGMbGzcqGH9aqpdaWcaaqaaiabdAeagnaaBaaaleaacqWGZbWCaeqaaOGaemiBaWgabaGaemOta4eaaiabc6caUaaa@353B@

If sampling is not equidistant, we can approximate the average *F*_*s *_as if the sampling was equidistant with help of the vectors *τ* and **t**

1Fs=τn−τn−1tn−tn−1=1N∑n=1N−1(τn−τn−1)
 MathType@MTEF@5@5@+=feaafiart1ev1aaatCvAUfKttLearuWrP9MDH5MBPbIqV92AaeXatLxBI9gBaebbnrfifHhDYfgasaacH8akY=wiFfYdH8Gipec8Eeeu0xXdbba9frFj0=OqFfea0dXdd9vqai=hGuQ8kuc9pgc9s8qqaq=dirpe0xb9q8qiLsFr0=vr0=vr0dc8meaabaqaciaacaGaaeqabaqabeGadaaakeaadaWcaaqaaiabigdaXaqaaiabdAeagnaaBaaaleaacqWGZbWCaeqaaaaakiabg2da9maalaaabaacciGae8hXdq3aaSbaaSqaaiabd6gaUbqabaGccqGHsislcqWFepaDdaWgaaWcbaGaemOBa4MaeyOeI0IaeGymaedabeaaaOqaaiabdsha0naaBaaaleaacqWGUbGBaeqaaOGaeyOeI0IaemiDaq3aaSbaaSqaaiabd6gaUjabgkHiTiabigdaXaqabaaaaOGaeyypa0ZaaSaaaeaacqaIXaqmaeaacqWGobGtaaWaaabCaeaacqGGOaakcqWFepaDdaWgaaWcbaGaemOBa4gabeaakiabgkHiTiab=r8a0naaBaaaleaacqWGUbGBcqGHsislcqaIXaqmaeqaaOGaeiykaKcaleaacqWGUbGBcqGH9aqpcqaIXaqmaeaacqWGobGtcqGHsislcqaIXaqma0GaeyyeIuoaaaa@5AE6@

where the quotient is a constant and independent of the index *n *(as long as the index exists).

## Authors' contributions

MA carried out the implementation of the methods, performed the computations and mainly drafted the manuscript. HL helped in developing the statistical methods and co-drafted the manuscript. AG provided the microarray measurement data and performed the Gene Set Enrichment Analysis. IS and OY-H conceived of the study and participated in its design and coordination. All authors read and approved the final manuscript.

## References

[B1] Robust regression for periodicity detection in non-uniformly sampled time-course gene expression data. Supplementary website. http://www.cs.tut.fi/sgn/csb/robustregper/.

[B2] Schena M, Shalon D, Davis R, Brown P (1995). Quantitative Monitoring of Gene Expression Patterns with a Complementary DNA Microarray. Science.

[B3] Wichert S, Fokianos K, Strimmer K (2004). Identifying periodically expressed transcripts in microarray time series data. Bioinformatics.

[B4] Chen J (2005). Identification of significant genes in microarray gene expression data. BMC Bioinformatics.

[B5] Ahdesmäki M, Lähdfdmäki H, Pearson R, Huttenen H, Yli-Harja O (2005). Robust detection of periodic sequences in biological time series. BMC Bioinformatics.

[B6] Fisher R (1929). Test of Significance in Harmonic Analysis. Proceedings of the Royal Society of London.

[B7] Glynn E, Chen J, Mushegian A (2005). Detection periodic pattersns in unevenly spaced gene expression time serises using Lomb-scargle periodogram. Bioinformatics.

[B8] Brockwell P, Davis R (1991). Time series: Theory and Methods.

[B9] de Lichtenberg U, Jensen L, Fausbø ll A, Jensen T, Bork P, Brunak S (2004). Comparisson of computational methods for the identification of cell cycle regulated genes. Bioinformatics.

[B10] Johansson D, Lindgren P, Berglund A (2003). A multivariate approach applied to microarray data for identification of genes with cell cycle-coupled transcription. Bioinformatics.

[B11] Liu D, Umbach D, Peddada S, Li L, Crockett P, Weinberg C (2004). A random-periods model for expression of cell-cycle genes. Proceedings of the National Academy of Sciences of the USA.

[B12] Lu X, Zhang W, Qin Z, Kwast K, Liu J (2004). Statistical resynchronization and Bayesian detection of periodically expressed genes. Nucleic Acids Research.

[B13] Luan Y, Li H (2004). Model-based methods for identifying periodically expressed genes based on time course microarray gene expression data. Bioinformatics.

[B14] Zhao L, Prentice R, Breeden L (2001). Statistical modeling of large microarray data sets to identify stimulusresponse profiles. Proceedings of the National Academy of Sciences of the USA.

[B15] Andersson C, Isaksson A, Gustafsson M (2006). Bayesian detection of periodic mRNA time profiles without use of training examples. BMC Bioinformatics.

[B16] Singh R, Palmer N, Gifford D, Berger B, Bar-Joseph Z (2005). Active Learning for Sampling in Time-Series Experiments With Application to Gene Expression Analysis. Proceedings of the 22nd International Conference on Machine Learning: Bonn, Germany.

[B17] Schwarzenberg-Czerny A (1996). Fast and statistically optimal period search in uneven sampled observations. The Astrophysical Journal.

[B18] Frick P, Baliunas S, Galyagin D, Sokoloff D, Soon W (1997). Wavelet Analysis of Stellar Chromospheric Activity Variations. The Astrophysical Journal.

[B19] Laguna P, Moody G, Mark R (1998). Power spectral density of unevenly sampled data by least-squareanalysis: performance and application to heart rate signals. IEEE Transactions on Biomedical Engineering.

[B20] Rasile M, Tagliaferri R, Milano L, Longo G (1997). Neural networks for periodicity analysis of unevenly spaced data. International Conference on Neural Networks.

[B21] Tarczynski A, Dongdong Q (2005). Optimal periodic sampling sequences for nearly-alias-free digital signal processing. IEEE International Symposium, on Circuits and Systems, 2005.

[B22] Tarczynski A, Bland D, Laakso T (1996). Spectrum estimation of non-uniformly sampled signals. Proceedings of the IEEE International Symposium on Industrial Electronics.

[B23] Bretthorst G, 1 (1988). Bayesian Spectrum, Analysis and Parameter Estimation.

[B24] Djurić P, Li HT (1995). Bayesian Spectrum Estimation of Harmonic Signals. IEEE Signal Processing Letters.

[B25] Qi Y, Minka T, Picard R (2002). Bayesian Spectrum Estimation of Unevenly Sampled Nonstationary Data. IEEE International Conference on Acoustics, Speech, and Signal Processing.

[B26] Zhou C, Wakefield J, Breeden L (2005). Bayesian Analysis of Cell-Cycle Gene Expression Data. UW Biostatistics Working Paper Series.

[B27] Hampel F, Ronchetti E, Rousseeuw P, Stahel W (1986). Robust statistics: The Approach Based on Influence Function.

[B28] Rousseeuw P, Leroy A (1987). Robust Regression and Outlier Detection.

[B29] Rousseeuw P, Van Aelst S, Van Driessen K, Gulló J (2004). Robust Multivariate Regression. Technometrics.

[B30] Tatum L, Hurvich C (1993). High Breakdown Methods of Time Series Analysis. Journal of the Royal Statistical, Society Series B (Methodological).

[B31] Duda R, Hart P, Stork D (2001). Pattern Classification.

[B32] ArrayExpress. http://www.ebi.ac.uk/arrayexpress/.

[B33] Huber P, Morgenthaler S, Ronchetti E, Stahel W, Basel (1993). Projection pursuit and robustness. New Directions in Statistical Data Analysis and Robustness.

[B34] Pearson R (2005). Mining Imperfect Data: dealing with contamination and incomplete records.

[B35] Subramanian A, Tamayo P, Mootha V, Mukherjee S, Ebert B, Gillette M, Paulovich A, Pomeroy S, Golub T, Lander E, Mesirov J (2005). Gene set enrichment analysis: A knowledge-based approach for interpreting genome-wide expression profiles. Proceedings of the National Academy of Sciences of the USA.

[B36] Kaleva O, Ihalainen H, Saarenrinne P (2000). A wavelet based method for the estimation of the power spectrum from irregularly sampled data. LADOAN10: 10–13 July 2000; Lisbon.

[B37] Klevecz R (2000). Dynamic architecture of the yeast cell cycle uncovered by wavelet decomposition of expression microarray data. Fund Integr Genomics.

[B38] Priestley M (1981). Spectral Analysis and Time Series.

[B39] Scargle J (1982). Studies in astronomical time series analysis. II. Statistical aspects of spectral analysis of unevenly spaced data. The Astrophysical Journal.

[B40] Benjamini Y, Hochberg Y (1995). Controlling the false discovery rate: a practical and powerful approach to multiple testing. J R Stat Soc, Ser B, Methodol.

[B41] Huber P (1981). Robust Statistics.

[B42] The MathWorks, Inc (2005). Statistics Toolbox User's Guide.

[B43] Rousseeuw P, Van Driessen K, Gaul W, Opitz O, Schader M (2000). An algorithm for positive-breakdown methods based on concentration steps. Data Analysis: Scientific Modeling and Practical Application.

[B44] Rousseeuw P, Van Driessen K (1999). A Fast Algorithm for the Minimum Covariance Determinant Estimator. Technometrics.

[B45] Schimmel M (2001). Emphasizing Difficulties in the Detection of Rhythms with Lomb-Scargle Periodograms. Biological Rhythm Research.

[B46] Kim H, Golub G, Park H (2005). Missing value estimation for DNA microarray gene expression data: local least squares imputation. Bioinformatics.

